# Global consensus on the management of melanin hyperpigmentation disorders

**DOI:** 10.1111/jdv.70185

**Published:** 2025-12-08

**Authors:** Thierry Passeron, Seemal R. Desai, Marwa Abdallah, Firas Al‐Niaimi, Ncoza Dlova, Pearl E. Grimes, Jorge Ocampo‐Candiani, Rashmi Sarkar, Leihong Flora Xiang, Helio Amante Miot

**Affiliations:** ^1^ Université Côte d'Azur Nice France; ^2^ University of Texas Southwestern Medical Center Dallas Texas USA; ^3^ Innovative Dermatology Plano Texas USA; ^4^ Ain Shams University Cairo Egypt; ^5^ Aalborg University Hospital Aalborg University Aalborg Denmark; ^6^ Taktouk Clinic London UK; ^7^ University of Kwa‐Zulu Natal Durban South Africa; ^8^ University of California, Los Angeles Los Angeles California USA; ^9^ Universidad Autonoma de Nuevo Leon Monterrey Mexico; ^10^ Lady Hardinge Medical College, Part of the Delhi University New Delhi Delhi India; ^11^ Huashan Hospital Fudan University Shanghai China; ^12^ Faculdade de Medicina UNESP Botucatu Brazil

**Keywords:** consensus development conference, cosmeceuticals, hyperpigmentation, melasma, periorbital hyperpigmentation, photoprotection, post‐inflammatory hyperpigmentation, skin pigmentation disorders, sunscreening agents, ultraviolet rays, visible light

## Abstract

Melanin hyperpigmentation disorders (MHD) are among the most prevalent dermatologic concerns globally, affecting individuals across all skin phototypes and geographies. Despite their widespread impact on quality of life—including stigmatization, anxiety and depression—comprehensive, unified and practical clinical guidelines are lacking. This global consensus project convened 10 leading dermatologists and pigmentary disorder experts from Africa, Asia, Europe and the Americas to develop an evidence‐based, internationally applicable framework for the management of common MHD, including melasma, post‐inflammatory hyperpigmentation, acquired dermal macular hyperpigmentation, periorbital hyperpigmentation, chronic hyperpigmentary photodamage, freckles/ephelides and hyperpigmentation of the folds. Based on a comprehensive literature review and a structured Delphi process, the expert panel formulated consensus statements and tailored treatment algorithms that account for skin type, photobiological behaviour, disease severity and regional access to therapies. Special emphasis was placed on the role of broad‐spectrum photoprotection and the challenges of tinted sunscreen availability in diverse populations. Treatment options ranged from topical lightening agents and systemic therapies (e.g. oral tranexamic acid) to procedural interventions (e.g. chemical peels, lasers and IPL) stratified by disease subtype and pigmentation depth. Notably, the consensus highlights that effective MHD management must address not only melanocytes but also the surrounding skin microenvironment, including fibroblasts, mast cells and vascular components. This consensus provides a globally adaptable clinical guide for the diagnosis and management of MHD, emphasizing a personalized approach that bridges scientific rigour with real‐world applicability across diverse populations and health systems.

## Graphical abstract

A global Delphi consensus of 10 dermatology experts established evidence‐based recommendations for melanin hyperpigmentation disorders, providing unified classification, photoprotection guidance and treatment algorithms. This framework harmonizes terminology and supports individualized management across diverse skin types and health systems.

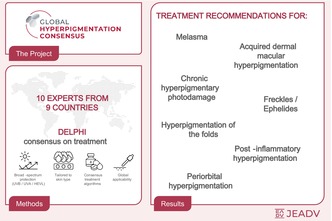

**Why was this study conducted?**

- Melanin hyperpigmentation disorders (MHDs) are highly prevalent worldwide and have a significant psychosocial impact, particularly among individuals with darker skin phototypes. However, no globally consistent, evidence‐based guidance exists to address the clinical variability, regional disparities and treatment challenges of these conditions across diverse populations and healthcare settings.

**What does this study add?**

- A global Delphi panel of 10 expert dermatologists established a consensus‐based classification and management of MHDs and recommendations on photoprotection tailored to skin type, and treatment algorithms considering pigmentation depth, clinical subtype and treatment availability.

**What are the implications of this article for disease understanding and/or clinical care?**

- This manuscript provides a globally applicable clinical framework that improves diagnostic precision, harmonizes terminology and guides individualized treatment of MHDs across diverse skin phototypes and geographies. It supports clinicians in resource‐limited settings and highlights areas for future research, contributing to equity in dermatologic care.

## INTRODUCTION

Pigmentary disorders are frequent dermatologic concerns worldwide, affecting all skin phototypes and significantly impacting quality of life (QoL), leading to stigmatization. A global survey on the six most common pigmentation disorders revealed a high self‐reported prevalence (50%) and a significant impact on quality of life, with 28% of participants scoring above 10 on the Dermatology Life Quality Index (DLQI) questionnaire. Social stigmatization was also considerable, with the highest scores observed for vitiligo, post‐inflammatory hyperpigmentation (PIH) and melasma. However, only 38% of respondents recognized sun exposure as harmful to their condition and practiced year‐round sun protection.^1^

Managing hyperpigmentation disorders poses challenges due to treatment resistance, recurrence and the risk of worsening hyperpigmentation with specific therapeutic modalities.^2^ Some hyperpigmentary conditions are particularly challenging to treat.^3^ These challenges can be attributed to a poorly understood pathophysiology, the multiplicity of underlying causes or the predominance of dermal melanin accumulation, preventing most topical formulations from reaching their target.^4^, ^5^, ^6^

Melanogenesis is a complex phenomenon involving multiple signalling pathways influenced by solar radiation, which affects skin pigmentation.^7^, ^8^ It is also affected by hereditary and exposome factors such as hormonal or metabolic disorders, other dermatological conditions, medications, pollution, stress and other external influences.^9^

In addition to photoprotection, melanin hyperpigmentation disorders (MHD) treatment modalities include topical cosmeceuticals, medical topical compounds, systemic drugs, chemical peels and interventions such as lasers, intense pulsed light (IPL) and radiofrequency. They should be tailored to the patient's hyperpigmentary disorder, skin type, genetic background, treatment availability and geographic location.

There is a lack of unified, globally applicable guidelines, as most publications emphasize local practices or single phototype contexts. Most publications address local or regional practices or focus on a particular phototype.^10^, ^11^ A global consensus on managing these disorders is needed, including an approach that considers skin type variations, genetic background, geographical location, solar irradiance and global treatment availability.

## METHOD

From September 2024 to April 2025, 10 experts in pigmentary disorders from Africa, Asia, Europe and the Americas gathered virtually and in person to develop an international, globally approached, evidence‐based framework for managing widespread pigmentary disorders, addressing regional differences and challenges involving skin types and treatment options. The panel of experts agreed to discuss photoprotection and seven hyperpigmentary disorders.

A comprehensive literature review established a foundation for the ensuing discussions. Each expert investigated their topic and shared the summaries for comments and feedback. Searches were performed across various databases, including PubMed, Medline, Google Scholar and Embase. The search terms utilized encompassed Photoprotection, Ultraviolet AND skin, Visible light AND skin, Melasma, Chloasma, Pregnancy Mask, Chronic Hyperpigmentary Photodamage, Solar lentigo, Actinic lentigo, Ephelides, Freckles, Post‐inflammatory Hyperpigmentation, Periorbital Hyperpigmentation, Dark Circles, Acquired Dermal Macular Hyperpigmentation, Lichen Planus Pigmentosus, Riehl's melanosis, Erythema Dyschromicum Perstans and Hyperpigmentation of the Folds. After filtering the results according to clinical studies, 152 relevant references were comprehensively reviewed to support this consensus.

This paper covers consensual approaches on photoprotection, melasma, chronic hyperpigmentary photodamage and freckles/ephelides, PIH, acquired dermal macular hyperpigmentation, hyperpigmentation of the folds and periorbital hyperpigmentation (POH).

When documented evidence was insufficient to settle an agreement, a modified Delphi method was used to design statements and treatment modalities matrices, seeking the experts' agreement.^12^ Statements were listed, and treatment options were recommended when 75% or more of the experts concurred with the proposed e‐survey options. All votes were anonymized through an electronic system. Statements and treatment options with less than 75% agreement among experts after the three Delphi rounds were considered not recommended. After each round, structured feedback was shared with the panellists, including anonymized summaries of the group responses. Panellists were then invited to reassess their answers in subsequent rounds, which is consistent with standard Delphi methodology.

Data collection was performed using a secure, web‐based electronic survey platform (Surveymonkey: https://www.surveymonkey.com/). This system ensured anonymity of responses, standardized data recording and facilitated iterative feedback between rounds.

The consensus was reached through two in‐person meetings and three virtual rounds of discussion.

## RESULTS

The raw data from the surveys are available at Data S1.

### Photoprotection

Modern understanding of skin photobiology supports current dermatological photoprotection recommendations. Solar irradiance fluctuates according to the seasons of the year and environmental parameters, including latitude, altitude, cloud cover and pollutants.^13^

Photoprotection is key in MHD. In addition to sun avoidance (seeking shade, wearing protective garments and using sunglasses), sunscreens provide essential protection. They are indicated for managing MHD and primary and secondary prevention. They must comply with broad‐spectrum protection, including filters for ultraviolet B (UVB) (with a Sun Protection Factor [SPF] of 50 or higher) and ultraviolet A (UVA) radiations.^14^, ^15^

Sunscreens that treat and prevent hyperpigmentary disorders must not only cover UVB but also longwave UVA and high‐energy visible light (HEVL).^16^, ^17^ In physiological conditions, visible light (VL) induces hyperpigmentation in Phototypes III–VI subjects but not in Phototype I and II subjects.^18^, ^19^, ^20^ In all skin types, HEVL can worsen MHD, such as melasma or PIH,^21^ by directly stimulating melanogenesis primarily through the OPSIN3 receptor and not by inducing oxidative stress,^7^ making the use of iron oxide or titanium dioxide the best option. Only these tinted sunscreens have demonstrated preventing VL‐induced hyperpigmentation in melasma and actinic lentigo in prospective randomized trials.^22^ Newer broad‐spectrum organic filters, such as phenylene bis‐diphenyltriazine (TriAsorB®), have also demonstrated protective activity against visible light.^23^, ^24^ Although interesting, these filters offer protection against the shorter wavelengths of visible light (400–420 nm) when wavelengths from 400 to 500 nm have also been shown to stimulate pigmentation.^25^ Antioxidants and plant polyphenols are incorporated into sunscreens and may reduce the immunosuppressive effects of UV radiation on the skin. They may also provide mild protection against VL, but cannot replace the use of substances that block VL.

Although the blue light emitted by TVs, computers and cell phones can stimulate pigmentation, its intensity is 100–1000 times lower than that of the sun. There has been no evidence that long‐term exposure to very low‐intensity blue light can stimulate pigmentation. A study by Duteil et al. has shown that exposure to blue light, using the maximum intensity emitted by the screens of actual devices for eight consecutive hours a day for 1 week, does not worsen melasma lesions.^26^ However, studies assessing the long‐term exposure are still required to definitively rule out the potential role of blue light emitted by device screens in worsening MHD.

The focus should be shifted from SPF (sun protection factor) to a more balanced UVB/UVA/VL protection. Real‐world conditions, like the amount of product reapplication, sweating and water exposure, should be considered in the effectiveness of sunscreens.^27^

Lighter skin tones need higher UVB protection. In the presence of MHD, darker and lighter skin tones need higher HEVL protection. But all phototypes are affected equally by UVA radiation and should be well protected from it, even if it is low‐energy radiation, as it accounts for 95% of all UV received. It is much more constant throughout the days and the year, and goes through the windows and clouds.^28^

Effectively utilizing sunscreens is also contingent upon affordability, application techniques and availability in different parts of the world. Researching the cost‐effectiveness of sunscreens is essential for advocacy.^27^

Photoprotection for melasma patients must include protection against HEVL.^19^ There is also interest in combining protection against UVB, UVA and shorter wavelengths of VL.^29^, ^30^

Using a broad‐spectrum UVB/UVA‐HEVL sunscreen (containing pigments) significantly decreased hyperpigmentation in actinic lentigo.^21^, ^31^, ^32^

Low doses of ambient light are sufficient to induce PIH.^33^ Areas wholly protected from dietary supplements may enhance photoprotection, but evidence is limited (Table 1).^34^

**TABLE 1:** Consensus statements with ≥75% agreement for photoprotection.

Photoprotection
Sunscreen formulations should include anti‐inflammatory and antioxidant additives to enhance efficacy for pigmentary conditions such as melasma and post‐inflammatory hyperpigmentation.
Indoor photoprotection is recommended for individuals exposed to direct sunlight through windows, particularly in cases of post‐inflammatory hyperpigmentation or melasma.
Sunscreen should be reapplied at least twice daily for individuals with regular outdoor exposure.
The amount of sunscreen applied should be enough to achieve an even, uniform layer (approximately one‐fourth teaspoon for the whole face).

In summary, photoprotection is key and mandatory in the care of all MHD. It relies not only on sunscreens but also on photoprotective habits. Non‐specialized physicians and patients need orientation on the correct quantity and type of sunscreen, and the impacts of weather and regional conditions. Optimal sunscreens for hyperpigmentary disorders must protect against UVB, UVA and HEVL.^25^, ^35^

Guidance on VL protection factor is still missing, and recommendations are being developed.^36^ Tinted sunscreens are only suitable for a small part of the population as there are limited colour options, and most of them are not ideal for all patients' needs, especially those with very light and dark skin types, and Asian skin type. Patients who are unfit to use available tinted sunscreens can apply a well‐balanced UVB/UVA non‐tinted sunscreen and add iron oxide‐containing makeup that matches their skin tone and protects against HEVL. The experts recommend developing affordable tinted sunscreens offering an extensive palette of colours to suit most skin tones.

Sunscreen products containing antioxidant, anti‐inflammatory and lightening ingredients are frequently recommended for treating melasma and PIH (Table 1). They also benefit other MHD where photoprotection is advised. However, the price of these products can be a barrier in low‐income countries.

It is advised that sunscreens be reapplied (approximately one‐fourth teaspoon for the whole face) during sun exposure; however, many patients do not adhere to this recommendation.^19^ Encouraging sunscreen application at least twice daily is essential for developing good habits, even in limited outdoor exposure.^37^ As the amount of sunscreen is linked to the level of photoprotection achieved, it should be sufficient to create an even, uniform layer (Table 1).

Oral photoprotection is not a consensus recommendation. Instead, it is now described as an adjunctive option suggested by some experts for managing pigmentary disorders, especially when combined with topical photoprotection. However, its efficacy is contingent on additional supportive evidence specific to diverse pigmentary conditions.

### Melasma

Melasma is a chronic skin condition that requires effective treatment, followed by maintenance therapy, to prevent relapse.^38^ Given the complex pathogenesis (involving keratinocytes, fibroblasts, mast cells and vascular components), its management often encompasses a combination of cosmeceutical, pharmaceutical and procedural therapies tailored to individual needs.^39^

Consistent and effective photoprotection, as previously described, is crucial to its management. Adequate sun protection is vital for preventing melasma, supporting treatment efforts and reducing the risk of relapses.^19^ Sunscreens should offer a high sun protection factor and robust protection against UVA1 and VL.^40^ Tinted sunscreens are an excellent choice, as pigments can guard against HEVL and UVA1 and may provide camouflage.

Therapeutic interventions include topical entities like lightening agents, antioxidants, exfoliants, moisturizers, anti‐angiogenesis agents and resurfacing procedures.^41^, ^42^, ^43^ Oral tranexamic acid may be used with adjuvant therapy to manage melasma. Resurfacing procedures such as chemical peeling, microneedling, laser treatments, energy‐based devices and platelet‐rich plasma may be used cautiously.

**FIGURE 1:**
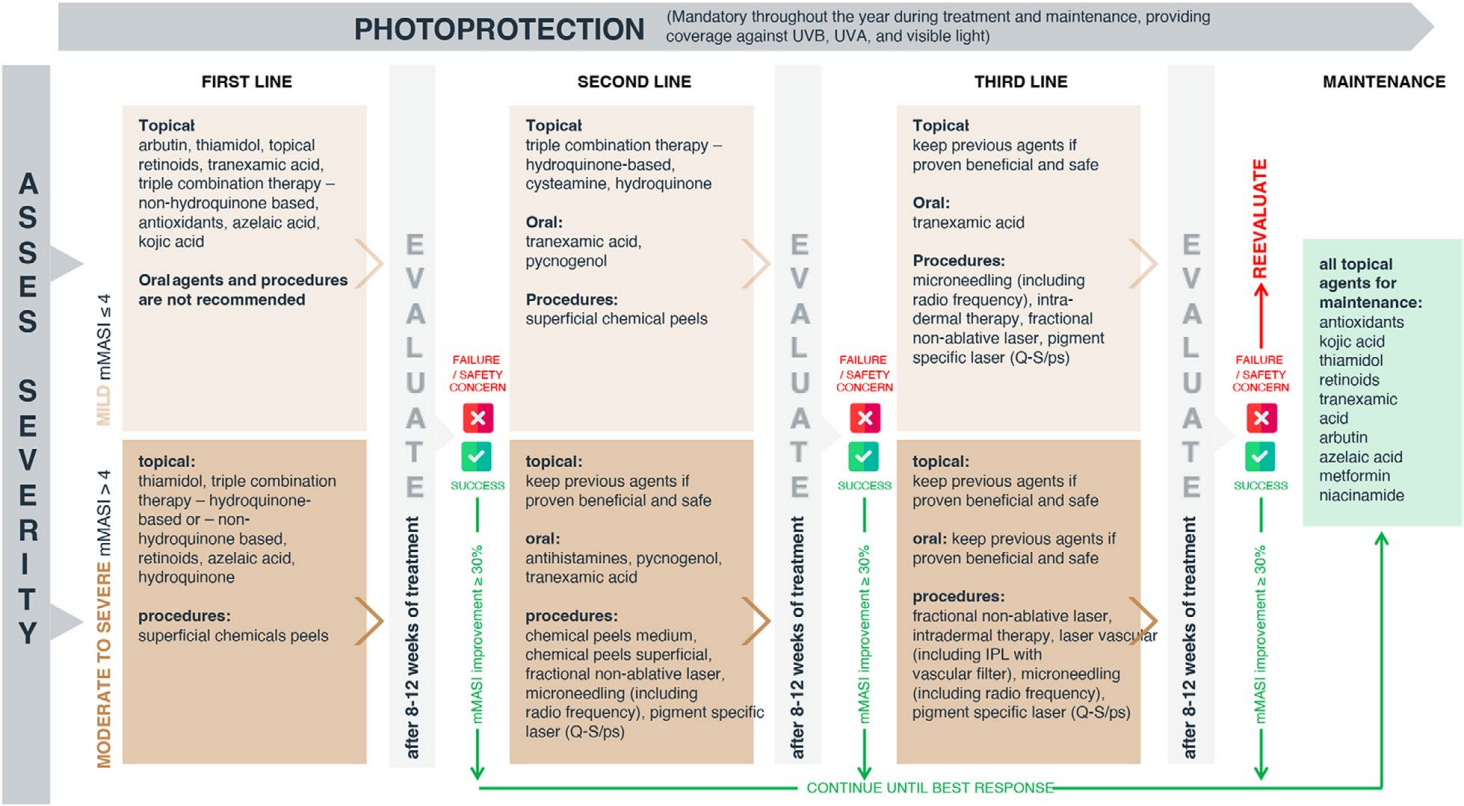
Consensual algorithm for the management of melasma. The recommended options for mild or moderate to severe melasma treatment are ordered by percentage of agreement and alphabetical order by each line of treatment and method of administration. The third‐line treatments recommended for mild melasma did not reach 75% agreement. IPL, intense pulsed light; mMASI, Modified Melasma Area and Severity Index; (Q‐S/ps), Q‐switched/picosecond; UVA, ultraviolet A radiation; UVB, ultraviolet B radiation.

Figure 1 summarizes the experts' consensus on the management of melasma. The experts defined patients with mild melasma as those with a mMASI ≤4. Patients with a mMASI >4 are considered to have moderate to severe melasma.^44^

Photoprotection is the mainstay of melasma prevention, management and maintenance, irrespective of the extent.

#### First‐line therapy

Established and novel topical agents can be used as monotherapy, combinations of individual agents or fixed triple combination therapies. Oral agents and procedures are not recommended as first‐line therapy for mild melasma, although they have a place in moderate to severe melasma.

#### Second‐line therapy

Depending on the therapeutic outcome of the first line, as assessed after 8–12 weeks and expected to achieve at least a 30% decrease in mMASI, more efficient and proven topical agents are recommended as second‐line therapy. Oral agents and procedures can be added accordingly.^44^

#### Third‐line therapy

Topical agents still show benefits and may be continued if proven beneficial and safe.^38^ Oral tranexamic acid is recommended if it has not been used previously or continues to show benefit with no significant adverse reactions in people with low thrombogenic risk.^45^ Microneedling and laser therapy can be used with great care to avoid worsening the condition.^38^, ^42^

Intravenous glutathione was evaluated as a potential option for melasma treatment. The experts agreed there is no place for this medication to manage melasma due to a lack of controlled data and major safety concerns.^46^

After determining the best response according to the severity of melasma and the type of treatment used, maintenance treatment is required to preserve and enhance the response, if attainable,^47^ but most importantly to prevent relapse. If clinically indicated, oral agents and procedures can be discontinued. Recommended topical agents resemble those listed for mild melasma. Hydroquinone‐based triple combination therapy should be avoided for maintenance.

New topical and systemic agents with multimodal mechanisms are available, allowing a shift towards innovative modalities.^47^, ^48^ Nonetheless, treatment selection should be personalized and depend on local availability.

### Chronic hyperpigmentary photodamage

Chronic exposure to sunlight leads to photoaging, which encompasses a variety of clinical manifestations, histological alterations and biochemical changes that distinguish it from the effects observed in chronologically aged skin that has been adequately sun protected.^49^

Chronic photodamage carries the risk of skin changes beyond pigmentation, including wrinkles, non‐melanoma skin cancer (NMSC) and lentigo maligna (LM).^50^, ^51^ A dermatologist's evaluation is essential to rule out cancer.^52^ Treatment targets include decreasing hyperpigmentary alterations (including pigment intensity and number of lentigos) and minimizing chronic erythema. Complete clearance is desirable, but often not possible.

Table 2 lists the statements that reached consensus. As treatment options, lasers, superficial chemical peels and cryotherapy reached unanimity. Lasers and light‐based devices like nanosecond, picosecond and IPL are effective for established and visible lentigos. They can be used as first‐line management, with caution exercised in higher skin types.

**TABLE 2:** Consensus statements with ≥75% agreement for chronic hyperpigmentary photodamage.

Chronic hyperpigmentary photodamage
Patch testing should not be performed for Poikiloderma of Civatte.
Topical retinoids can be proposed as the first‐line and long‐term maintenance for photoaging.
Topical therapy alone can be sufficient for light or non‐border‐formed lentigos.
Lasers and/or chemical peels (superficial to medium depth) are recommended as the first‐line treatment for pigment changes resulting from chronic photodamage.
Lasers are more effective than cryotherapy or topical treatments for established visible lentigos in fair skin types.

Superficial and medium chemical peels were recommended as alternatives to lasers and can be used as first‐line options.

Higher recurrence rates and the risk of dyschromia limit the use of cryotherapy.

Topical depigmenting agents were also recommended. They may help reduce pigmentation, are adequate for preparation and can be sufficient for light or non‐border‐formed lentigos. Retinoids can be used as first‐line therapy and for maintenance.

Monitoring the affected areas is not mandatory and is contingent upon the patient's condition. Annual follow‐ups or subsequent assessments should be conducted if NMSC results from chronic photodamage.

The efficacy of treatment is generally assessed at a 3‐month post‐treatment point.^53^ In the case of Poikiloderma of Civatte, long‐term follow‐up or monitoring is not required after treatment, and patch testing for this condition should not be performed.

Intense pulsed light and short wavelengths (532 nm) should be used with caution in darker skin types (Fitzpatrick V–VI) to prevent PIH.

The prevention of photodamage's initiation or perpetuation is of utmost significance and benefit by thoroughly elucidating the underlying mechanisms involved in this process. Current strategies encompass rigorous photoprotection, the use of antioxidants and brightening agents, which may be further enhanced by annual chemical peel or laser therapy procedures.

### Freckles/Ephelides

Freckles, also known as ephelides, are localized areas of hypermelanosis caused by a mosaic distribution of melanocytes with diverse melanocortin 1 receptor (MC1R) polymorphisms, resulting in varied skin responses to UV radiation.^54^, ^55^ They primarily appear in sun‐exposed areas in children and young individuals with fair skin, gradually becoming less prominent with age. In regions with distinct seasons, freckles tend to darken during summer and fade in winter.

Freckles are recognized as indicators of increased susceptibility to early photoaging and a heightened risk of skin cancer.^56^ It is essential to differentiate them from nevi, lentigines and early melanoma.

Table 3 shows consensus recommendations for freckle treatment. Most clinical trials consider a 50–75% reduction in the lesions as a significant outcome.^57^, ^58^ Treatment follow‐up with strict photoprotection is crucial.

**TABLE 3:** Consensus statements with ≥75% agreement for freckle treatment and maintenance.

Treatment options	Maintenance strategies
Intense pulsed light Q‐switched/pico (532, 694 and 755)	Topical retinoids

Experts recommend implementing maintenance treatments using topical retinoids (Table 3) and/or tyrosinase inhibitors.

Additionally, periodic follow‐up consultations are recommended to adjust treatments based on tolerability and enable early management of relapses. Patient education on the condition's natural course, photoprotection and the consistent use of topical depigmenting agents and/or retinoids is essential to prevent recurrence.

Table 4 summarizes the agreed consensus statements for post‐inflammatory hyperpigmentation, periorbital hyperpigmentation / dark circles, acquired dermal macular hyperpigmentation and hyperpigmentation of the folds.

**TABLE 4:** Consensus statements with ≥75% agreement for post‐inflammatory hyperpigmentation, periorbital hyperpigmentation/dark circles, acquired dermal macular hyperpigmentation and hyperpigmentation of the folds.

Post‐inflammatory hyperpigmentation
For patients at higher risk of developing PIH, it is highly recommended to prepare the skin before performing procedures to minimize the risk of PIH.
Pre‐treatment with skin‐lightening agents improves the efficacy of procedural therapies for PIH.
Some cases of PIH do not require immediate treatment and can be managed by waiting.
In the case of treatment, topical therapy should be the first line of treatment for PIH, with procedures like lasers and peels reserved for second‐line or recalcitrant cases.

Periorbital hyperpigmentation/Dark circles
Eyelid stretch tests can be used as a routine tool for diagnosing POH.
Dermoscopy can be used as a routine tool for diagnosing POH.
Periodic dermoscopic imaging can be used to track treatment progress.

Acquired dermal macular hyperpigmentation
A unified nomenclature for LPP, Riehl's melanosis, and EDP under ADMH is essential for standardizing diagnosis and management.
Contaminants such as allergens may play a significant role in the aetiology of ADMH and should be addressed during patient evaluation.
Patch testing (including photopatch) is recommended for Riehl's melanosis.
Pre‐treatment with topical or oral agents is recommended before initiating procedural treatments for LPP or Riehl's melanosis.
Lasers or IPL should be avoided during the active inflammatory stage of LPP to prevent exacerbation.

Hyperpigmentation of the folds
Assessment with Wood's Lamp is recommended to exclude Erythrasma.
Non‐fluorinated topical steroids can be limited to short‐term use only.

Abbreviations: ADMH, acquired dermal macular hyperpigmentation; EDP, erythema dyschromicum perstans; IPL, intense pulsed light; LPP, lichen planus pigmentosus; PIH, post‐inflammatory hyperpigmentation; POH, periorbital hyperpigmentation.

### Post‐inflammatory hyperpigmentation

PIH may appear following any inflammatory skin condition (e.g. infectious or inflammatory skin dermatoses and hypersensitivity reactions to medications). PIH may have a genetic predisposition,^59^ occurs at any age, in any skin phototype (although most common in skin of colour) and is mainly related to acne, post‐procedural (peeling, laser), atopic dermatitis and impetigo.^60^

Following inflammation, melanin production increases or is abnormally deposited in the epidermis and/or dermis. Inflammatory mediators thought to be related include prostaglandins (PG), such as PGE2, leukotrienes (LT), including LTC4 and LTD4, cytokines like interleukin‐33 (IL‐33) and others, which have been shown to stimulate epidermal melanocytes. In turn, a basal membrane disruption is noted with dermal deposition of melanin and subsequent macrophage activation.^60^, ^61^

Table 5 summarizes the consensus reached on the management of PIH.

**TABLE 5:** Consensus statements with ≥75% agreement for post‐inflammatory hyperpigmentation: Recommended treatment options and prevention measures.

Treatment		
	Acne^a^ PIH	Non‐acne^b^ PIH
Topical	Thiamidol Azelaic acid Topical retinoids Hydroquinone Tranexamic acid	Hydroquinone Thiamidol Azelaic acid Tranexamic acid (topical/oral)^c^ Cysteamine Triple combination Topical retinoids
Procedures	Chemical peels (e.g. glycolic acid, TCA)	Chemical peels (e.g. glycolic acid, TCA) Low fluence Q‐switched Nd:YAG laser
**Prevention**		
Avoidance of irritants (e.g. retinoids close to procedures) Skin priming with lightening agents		

*Note*: Treatment options for acne and non‐acne PIH are ordered by percentage of agreement and alphabetical order for topical agents and procedures.

Abbreviations: PIH, post‐inflammatory hyperpigmentation; TCA, trichloroacetic acid.

^a^ Hyperpigmentation secondary to acne.

^b^ Hyperpigmentation due to other inflammatory skin conditions like eczema, burns or aggressive skin treatments.

^c^ Tranexamic acid may be used either topically or orally, depending on the treatment stage.

PIH management entails treating the underlying dermatoses while simultaneously stressing the importance of accompanying photoprotection.^62^ The goal of treating PIH is to reduce inflammation while lightening the pigmented skin with these combinations. Effective treatment can be challenging, and ablative interventions can potentially worsen the condition.^63^

The depth of the pigment in the skin determines its appearance and the treatment options available. Epidermal PIH appears as a brown discoloration, while dermal PIH occurs deeper in the dermis, presenting as a more blue‐grey hue and is often more challenging.

Treatment also depends on the aetiology of PIH (Table 5). ‘Acne’ PIH refers to hyperpigmentation secondary to acne, while ‘non‐acne’ PIH is due to other inflammatory skin conditions like eczema, burns or aggressive skin treatments.

Topical therapy is considered the first‐line treatment option for PIH. Table 2 depicts the recommendations made by the experts, irrespective of the primary aetiology.

Procedural‐based therapies may be used in cases of PIH but are usually not considered first‐line. Options include in‐office treatments such as chemical peels and low‐fluence Q‐switched Nd:YAG Laser (non‐acne PHI). Care must be taken when treating patients with procedural‐based therapies to ensure that the treatment itself does not induce skin damage, further hyperpigmentation or exacerbate the preceding PIH.

Oral tranexamic acid, effective in melasma, has been proposed in PIH,^64^ but more data are needed.

A comprehensive understanding of surgical and non‐surgical techniques, as well as proper skin preparation with skin lighteners or anti‐inflammatory agents before therapeutic procedures, is crucial to minimize side effects, such as PIH, especially in individuals with a high Fitzpatrick phototype.^62^, ^65^

Depending on the underlying cause, PIH may improve without intervention.^60^ In those circumstances, appropriate patient guidance on the aetiology and potential aggravating or alleviating factors should be offered.

### Periorbital hyperpigmentation

POH is a global challenge. It is also known as periorbital melanosis, dark circles, under‐eye circles, infraorbital melanosis or idiopathic chronic hyperpigmentation of the orbital region.^66^, ^67^, ^68^ It includes the upper and lower eyelids, particularly the lower ones. Although a familial component has been identified, it affects all genders. Still, it is more frequent in females and SOC.^69^, ^70^ Due to its complex pathogenesis, no straightforward therapeutic options are available and a proper diagnosis of the aetiology of POH is mandatory before proposing any care.

POH is classified as follows: Constitutional—a dark curved band appearing on the lower eyelids, resembling the orbital rim and often affecting the upper eyelids. Post‐inflammatory—brownish or grey eyelid patches sometimes showing lichenification, accentuated skin creases and nearby eczematous papules. A personal or family history of atopy may be present. Vascular—primarily impacts lower eyelids, showing prominent capillaries or telangiectasia and bluish discoloration. Visible veins become more apparent with skin stretching, creating dark circles due to skin transparency and dermal vascularity. Shadow effect—Dark shadow under the tarsal muscle, eye bags or deep tear trough on the medial orbital rim that disappears with direct light.^69^

**FIGURE 2:**
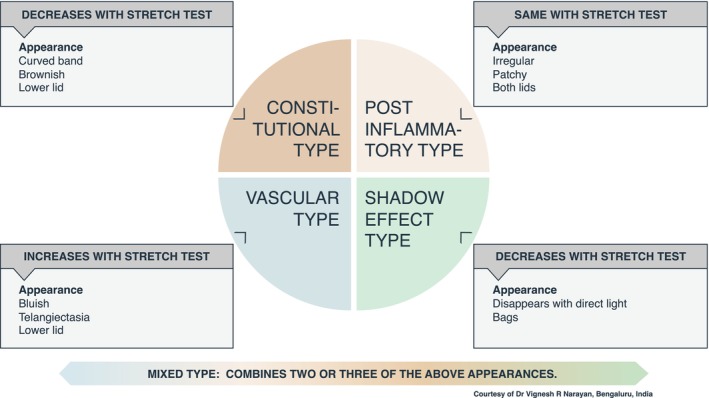
Periorbital hyperpigmentation: Main characteristics of the subtypes.

Eyelid stretch tests and dermoscopy are recommended as standard diagnostic tools for assessing POH (Figure 2). The stretch test helps differentiate between true pigmentation, vascular component and shadowing effect by gently pulling on the lower eyelid. With pigmentation, the colour remains unchanged when stretched. The colour improves or completely disappears with vascularity, and the vessels become more apparent.^71^ Dermoscopy is a non‐invasive diagnostic technique that reveals structures in the skin not visible to the naked eye, aiding in determining the cause, whether due to pigmentation, underlying vasculature or both.^72^ As the treatment depends on aetiology, dermoscopic imaging should be employed throughout the follow‐up period to monitor treatment advancements, though it does not provide quantitative data (Table 6).

**TABLE 6:** Consensus statements with ≥75% agreement for periorbital hyperpigmentation: Recommended management per type.

	Constitutional	Post‐inflammatory	Vascular	Shadow effect
Topical	Azelaic acid Thiamidol	Azelaic acid Vitamin C Thiamidol Arbutin Hydroquinone Polyphenols Niacinamide	Caffeine^a^ Vitamin C	None
Procedures	Camouflage			
	Chemical peels Laser therapy	Chemical peels	Laser therapy	Dermal fillers^b^

*Note*: Treatment options for each type of periorbital hyperpigmentation are ordered by percentage of agreement and alphabetical order for topical agents and procedures.

^a^ Caffeine is only to be used for the vascular type.

^b^ Dermal fillers are only to be used for the shadow effect type.

Table 6 lists the recommended POH management. For the constitutional type, azelaic acid and thiamidol were recommended, along with chemical peels and laser therapy.

Azelaic acid, vitamin C, thiamidol, arbutin, hydroquinone, polyphenols and niacinamide were recommended for the post‐inflammatory type, as were chemical peels and laser therapy.

Caffeine and vitamin C were recommended for the vascular type, along with laser therapy.

No surveyed topical agent reached the 75% agreement for the shadow effect type. Dermal fillers are the recommended procedure for this condition.

Caffeine was only recommended for the vascular type, and dermal fillers were only for the shadow effect type. Camouflage is a procedure recommended for all types of POH.

### Acquired dermal macular hyperpigmentation

ADMH is a recently established term^73^ encompassing heterogeneous yet overlapping entities of ‘acquired’ origin, ‘dermal’ localization and ‘macular’ patterns of ‘hyperpigmentation’ evidenced by histopathological and clinical examinations. This term unifies disorders known as Riehl's melanosis, lichen planus pigmentosus (LPP) and erythema dyschromicum perstans (EDP). It helps to standardize diagnoses and treatment protocols (Table 4). These disorders exhibit significant clinical and pathological similarities and a poor response to conventional treatments. Early diagnosis of ADMH poses a significant clinical challenge,^74^, ^75^ as non‐specialized physicians may misdiagnose it as PIH or other hyperpigmentary disorders. Unfortunately, a longer disease course may lead to poorer therapeutic outcomes.

As indicated in Table 1, allergens and other contaminants can significantly influence the causes of ADMH and should be considered when evaluating patients. The association with autoimmune diseases beyond LPP remains unclear.^76^ Further clinical studies are required to determine whether routine screening for antinuclear antibodies and other autoantibodies is warranted.

Different entities in this group present specific handling. Patch testing, including photopatch testing, is often conducted for suspected Riehl's melanosis (Table 4). However, the appropriateness of such testing for LPP remains debatable, and it is not recommended for EDP. Access to specialized patch testing and regulatory guidelines varies across different geographic locations. Further clinical research is needed to identify high‐risk allergens associated with ADMH and develop disease‐specific diagnostic kits to facilitate precision screening and clinical management.

Oral (e.g. topical calcineurin inhibitors) and topical agents should be proposed before considering procedural treatments for LPP or Riehl's melanosis (Table 4). During the acute inflammatory phase of LPP, it is advisable to refrain from using lasers or IPL treatments to prevent exacerbation. Future studies on lasers should focus on standardizing patient selection, criteria for choosing a laser type and standardizing laser protocols and parameters.

In the context of ADMH, the cosmeceuticals utilized include allergen‐free emollients and moisturizers. Additionally, broad‐spectrum sunscreens are recommended for individuals with photosensitive skin. Furthermore, skin‐lightening products containing ingredients such as vitamin C, niacinamide, tranexamic acid and arbutin, among others, may be employed as a second‐line treatment option.

Table 7 presents the recommended treatments for each of the ADMH disorders. The listed topical agents, oral medications and procedures reached the experts' agreement for Riehl's melanosis, LPP and EDP, respectively.

**TABLE 7:** Consensus statements with ≥75% agreement for acquired dermal macular hyperpigmentation: Recommended treatments per disorder.

Riehl's melanosis	Lichen planus pigmentosus	Erythema dyschromicum perstans
Topical calcineurin inhibitors (e.g. tacrolimus) Oral isotretinoin Topical steroids Lasers (e.g. Q‐switched Nd:YAG, IPL)	Topical calcineurin inhibitors (e.g. tacrolimus) Oral isotretinoin Topical Steroids Oral steroids Hydroxychloroquine Topical JAK inhibitor Lasers (e.g. Q‐switched Nd:YAG, IPL)	Oral isotretinoin Topical calcineurin inhibitors (e.g. tacrolimus) Topical steroids Oral steroids Hydroxychloroquine Lasers (e.g. Q‐switched Nd:YAG, IPL)

*Note*: Treatment options for each disorder are ordered by percentage of agreement and alphabetical order for topical and oral agents and procedures.

Abbreviation: IPL, intense pulsed light.

Future research endeavours may investigate treating this condition using a combination of strategies, including topical therapies, oral pharmacological agents and laser interventions. Developing standardized therapeutic protocols is imperative to facilitate clinically meaningful improvements for affected individuals, enhancing their overall QoL.

It is recommended that the ADMH Dermal Pigmentation Area and Severity Score (DPASI) be routinely used in all studies on ADMH.^77^ Consider that DPASI provides a standardized assessment for facial and neck involvement but has limitations when evaluating LPP and EDP cases with truncal and extremity involvement. Future development of a comprehensive scoring system encompassing truncal and extremity involvement would be valuable.

### Hyperpigmentation of the folds

HF is a common skin condition often seen in individuals with darker skin tones (Phototypes III–V). Its pathophysiology is largely unknown; PIH has been suggested as a potential explanation. It may result from irritation or trauma, leading to darkened patches on the affected skin.^78^

A differential diagnosis should be considered with other hyperpigmented dermatoses that include predominant flexural pigmentation like erythrasma, acanthosis nigricans, atopic dermatitis, terra firma‐forme dermatosis, confluent and reticulated papillomatosis, Dowling‐Degos disease, dyskeratosis congenita, flexural pigmentation with multiple lentigines, Galli‐Galli disease, granular parakeratosis, Harber's syndrome, certain infections and neurofibromatosis (Crowe's sign). PIH, particularly from contact dermatitis, is also a common cause. Additionally, systemic conditions like Addison's disease and hemochromatosis can also manifest with hyperpigmentation in these areas.

Aside from physical factors resulting from hair removal or friction, such as tight clothing, chemical substances applied to the region, including cleansing products or antiperspirants, can be identified through patch testing. Patch testing is not recommended for all cases, but only for those where irritants or allergens are suspected of triggering hyperpigmentation of the folds.

Specific agents may cause discomfort in hot climates, particularly in body areas prone to heat accumulation.

Hyperpigmentation of the folds (HF) is mainly treated with depigmenting agents (Table 8). The recommended agents include thiamidol, niacinamide, topical retinoids, hydroquinone, short courses of desonide (0.05%), glycolic acid (10–20%) and urea (20%). Nonetheless, these treatments often show inconsistencies and may cause skin irritation. Hydroquinone and non‐fluorinated steroids like desonide should only be used for the short term (2–4 weeks), with limited application time (~30 min) and frequency (twice per week; Table 1) Topical treatments can be combined with procedures such as superficial chemical peelings and low‐fluence Q‐switched Nd:YAG laser.

**TABLE 8:** Consensus statements with ≥75% agreement for hyperpigmentation of the folds: Recommended treatment options and prevention measures.

Treatment	Prevention
Topical agents Thiamidol Niacinamide Topical retinoids Hydroquinone Desonide (0.05%) Glycolic acid (10–20%) and urea (20%) Procedures Superficial peelings Q‐switched Nd:YAG laser	Avoidance of irritants and allergens Avoidance of trauma‐inducing procedures (waxing, tweezing)

*Note*: Treatment options are ordered by percentage of agreement and alphabetical order for topical agents and procedures.

The primary prevention strategies unanimously recommended include avoiding irritants, allergens and trauma‐inducing procedures such as waxing and tweezing.

## PERSPECTIVES

Melanin hyperpigmentary disorders are a complex and heterogeneous group that requires a precise diagnosis before proposing any treatment.

The challenges in managing pigmentary dermatoses lie in developing sunscreens with broader radiation coverage, greater efficacy for daily use and cosmetics suitable for different skin types, considering that most users apply insufficient amounts and do not reapply the products. Additionally, developing topical lightening agents with high efficacy and cosmetic tolerability, suitable for different skin types and varying sun exposure conditions, that deliver rapid and long‐lasting results, remains a field of investigation.

Significant advances have been made in producing cosmetic lightening agents, some of which are at least as effective as hydroquinone. However, most current approaches target the melanocytes while the impact of the surrounding keratinocytes and the key role of the dermal component (including fibroblasts, endothelial cells, sebocytes and mastocytes' secreted factors) are increasingly being underlined. Developing compounds that prevent the release of these factors or their binding to melanocyte receptors appears to be mandatory for achieving optimal efficacy. Targeting additional pathways recently reported to be involved in some melanin hyperpigmentary disorders offers exciting perspectives, such as modulating epigenetic modifications in melanocytes or fibroblasts, combating oxidative stress (including mitochondrial oxidative stress) and stimulating autophagy. Finally, developing new devices to reach and modulate the dermal component without harming the epidermal cells could provide long‐term efficacy for these disorders.

## AUTHOR CONTRIBUTIONS

Conceptualization: T. Passeron, S.R. Desai, M. Abdallah, F. Al‐Niaimi, N. Dlova, P. E. Grimes, J. Ocampo‐Candiani, R. Sarkar, L. F. Xiang and H. A. Miot. Methodology: T. Passeron and H. Miot. Supervision: T. Passeron and H. Miot. Writing (original draft/review and editing): T. Passeron, S.R. Desai, M. Abdallah, F. Al‐Niaimi, N. Dlova, P. E. Grimes, J. Ocampo‐Candiani, R. Sarkar, L. F. Xiang and H. A. Miot.

## FUNDING INFORMATION

Editorial assistance was provided by Beiersdorf. The sponsor had no role in the study design, data collection, analysis, interpretation or decision to submit the manuscript. These tasks were solely the responsibility of the authors.

## CONFLICT OF INTEREST STATEMENT

Thierry Passeron: Consulting honoraria from Almirall, AbbVie, BMS, Incyte, Janssen, Lilly, Novartis, Pfizer, UCB and Vyne Therapeutics. Payment or honoraria for lectures, presentations, speakers bureaus, manuscript writing or educational events from Almirall, AbbVie, Amgen, BMS, Celgene, Galderma, GSK, Incyte, Janssen, LEO Pharma, Lilly, MSD, Novartis, Pfizer, Sanofi, SUN Pharma, Takeda and UCB. Support for attending meetings and/or travel from AbbVie, Incyte, Janssen, Lilly, Novartis and UCB. Institutional patents planned, issued or pending: WNT agonists and GSK3 inhibitors in vitiligo, CXCR3B in vitiligo and quinoline compounds in solid cancers. Seemal R. Desai: Consulting honoraria from Beiersdorf, Pfizer and Incyte, and has held multiple dermatology leadership positions, as well as clinical research and consulting work. Marwa Abdallah: Consulting honoraria from Isispharma, Beiersdorf, Bayer, Janssen, AbbVie and Pfizer. Firas Al‐Niaimi: Consulting honoraria from Beiersdorf. Ncoza Dlova: Received honorarium and/or conducted clinical trials from Beiersdorf, L'Oréal, Pierre Fabre, Sanofi and Aventis. Pearl E. Grimes: Consulting honoraria from Incyte, AbbVie, Pfizer and Lilly. Jorge Ocampo‐Candiani: Advisory, speaker, honoraria from Beiersdorf, Isdin, L'Oréal and Pierre Fabre. Institution, investigator honoraria from Abbott, Anacor Pharmaceuticals, Bristol Myers Squibb, Glaxo Smith Kline, Janssen, Novartis, Sanofi‐Aventis Recherche & Développement, Eli Lilly and Company, BOEHRINGER INGELHEIM/CERNER ENVIZA, Pfizer Inc., Merck Sharp & Dohme LLC, DermBiont, Inc. and Incyte Corporation. Rashmi Sarkar: Declares no conflicts of interest. Leihong Flora Xiang: Consultant to L'Oréal, Beiersdorf, Botanee, JALA and Estée Lauder. Hélio A. Miot: Advisory and speaker from Galderma, Kenvue, L'Oréal, Pfizer, Theraskin and Beiersdorf. Investigator honoraria from AbbVie, Pierre‐Fabre, Galderma and Theraskin.

## ETHICAL APPROVAL

Not applicable. This study did not involve human participants, tissue or data.

## ETHICS STATEMENT

Ethical assessment was not required, as no patient data were analysed in this study.

## Supporting information


Data S1.

## Data Availability

Data sharing is not applicable to this article as no new data were created or analyzed in this study.
