# Supplementary material for: Global consensus on the management of melanin hyperpigmentation disorders
Source: J Eur Acad Dermatol Venereol. 2025 Dec 8;40(5):760–72. doi: 10.1111/jdv.70185 (PMC13109754; doi:10.1111/jdv.70185)
Supplement: Supplementary file 1 — Data S1. [file JDV-40-760-s001.docx]

**Global Consensus on the Management of Melanin Hyperpigmentation Disorders.**

| **Photoprotection** |  |
| --- | --- |
| Oral photoprotection supplements (e.g., *polypodium leucotomos*, pycnogenol) should be considered as part of the management plan for pigmentary disorders. | AGREEMENT |
|  | 50% |
| Photoprotection is necessary even indoors for people suffering of melanin hyperpigmentary disorders. | AGREEMENT |
|  | 60% |
| Sunscreen formulations should include anti-inflammatory and antioxidant additives to improve efficacy for pigmentary conditions like melasma and PIH. | AGREEMENT |
|  | 80% |
| The amount of sunscreen applied should be enough to achieve an even, uniform layer (approximately ¼ teaspoon for the whole face) | AGREEMENT |
|  | 100% |
| What should be the recommended amount of sunscreen to be applied? | AGREEMENT |
| 1 teaspoon | 70% |
| Applying twice daily | 80% |
| Applying 3 times daily | 20% |
| Applying every 2h | 20% |
|  |  |

| **Hyperpigmentary chronic photo damage** |  |
| --- | --- |
| Lasers and chemical peels should be recommended as the first-line treatment for hyperpigmentary chronic photo damage. | AGREEMENT |
|  | 80% |
| Topical therapy alone can be sufficient for light or non-border-formed lentigo. | AGREEMENT |
|  | 100% |
| IPL (Intense Pulsed Light) and short-wavelength (532nm) treatments should be avoided in individuals with darker skin types (Fitzpatrick IV-VI) and those of Asian ancestry to minimize the risk of post-inflammatory hyperpigmentation (PIH). | AGREEMENT |
|  | 70% |
| Topical retinoids can be proposed for the long-term management of photoaging. | AGREEMENT |
|  | 100% |
| AL - Lasers are more effective than cryotherapy or topical treatments for active lentigo in fair skin types. | AGREEMENT |
|  | 90% |
| AL - Chemical peels are a suitable alternative where laser treatments are not accessible. | AGREEMENT |
|  | 100% |
| AL - Topical retinoids should be recommended as the primary treatment for photodamage, associated with photoaging improvements. | AGREEMENT |
|  | 90% |
| PC - Oral tranexamic acid is effective for treating vascular and pigmented components of severe photodamage. | AGREEMENT |
|  | 40% |
| Patch testing is required for Poikiloderma of Civatte. | AGREEMENT |
|  | 10% |

**Freckles Treatment Options**

| Treatment | Recommendation |
| --- | --- |
| Intense Pulsed Light (IPL) | 80% |
| Q-Switched / pico (532, 694, and 755) | 80% |
| Topical Depigmenting Agents | 70% |
| Superficial Chemical Peels (Useful for exfoliation and depigmentation) | 70% |
| Triple Combination | 50% |
| Medium Chemical Peels | 20% |
| Deep Chemical Peels (Reserved for recalcitrant cases) | 0% |

Freckles Maintenance Strategies

| Treatment | Recommendation |
| --- | --- |
| Topical Retinoids | 80% |
| Topical Depigmenting Agents | 70% |
| Periodical Chemical Peelings | 50% |
| Periodical Therapy Laser Treatment | 40% |

**Mild Melasma Algorithm**

1st line of treatment

| Agent | Yes |
| --- | --- |
| Arbutin | 100% |
| Thiamidol | 100% |
| Topical Retinoids | 100% |
| Tranexamic Acid (Topical) | 90% |
| Triple Combination Therapy – non-hydroquinone based | 90% |
| Antioxidants (e.g., Vitamin C) | 80% |
| Azelaic Acid (20%) | 80% |
| Kojic Acid | 80% |
| Metformin | 70% |
| Niacinamide | 70% |
| Hydroquinone (4%) | 60% |
| Triple Combination Therapy – hydroquinone-based | 60% |
| Malassezin | 60% |
| Cysteamine | 50% |
| Methimazole | 40% |
| 2MNG | 40% |
| Neotone | 30% |
| Chemical Peels superficial | 20% |
| Melatonin (oral) | 10% |
| Laser vascular (including IPL with vascular filter) | 10% |
| Microneedling (including radio frequency) | 10% |
| Intradermal therapy | 10% |
| Iontophoresis | 10% |
| Tranexamic Acid (Oral) | 0% |
| Chemical Peels medium | 0% |
| Chemical Peels deep | 0% |
| Pycnogenol (oral) | 0% |
| Anti-histamine (oral) | 0% |
| Pigment-specific laser (Q-S / ps) | 0% |
| Fractional ablative laser | 0% |
| Fractional non-ablative laser | 0% |
| PRP | 0% |
| Glutathione (oral) | 0% |
| Glutathione IV | 0% |

Mild Melasma Algorithm

2nd line of treatment

| Agent | Yes |
| --- | --- |
| Chemical Peels superficial | 100% |
| Tranexamic Acid (Oral) | 100% |
| Triple Combination Therapy – hydroquinone-based | 90% |
| Cysteamine | 80% |
| Hydroquinone (4%) | 80% |
| Pycnogenol (oral) | 80% |
| Thiamidol | 70% |
| Chemical Peels medium | 70% |
| Anti-histamine (oral) | 70% |
| Azelaic Acid (20%) | 60% |
| Triple Combination Therapy – non-hydroquinone based | 60% |
| Antioxidants (e.g., Vitamin C) | 60% |
| Topical Retinoids | 60% |
| Metformin | 60% |
| Microneedling (including radio frequency) | 60% |
| Tranexamic Acid (Topical) | 50% |
| Kojic Acid | 50% |
| Neotone | 50% |
| Niacinamide | 50% |
| Intradermal therapy | 50% |
| 2MNG | 40% |
| Arbutin | 40% |
| Melatonin (oral) | 40% |
| Laser vascular (including IPL with vascular filter) | 40% |
| Malassezin | 30% |
| Methimazole | 30% |
| Pigment specific laser (Q-S / ps) | 30% |
| Fractional ablative laser | 30% |
| PRP | 30% |
| Glutathione (oral) | 30% |
| Fractional non-ablative laser | 20% |
| Iontophoresis | 20% |
| Chemical Peels deep | 0% |
| Glutathione IV | 0% |

Mild Melasma Algorithm

3rd line of treatment

| agent | Yes |
| --- | --- |
| Microneedling (including radio frequency) | 100% |
| Intradermal therapy | 90% |
| Fractional non-ablative laser | 80% |
| Pigment specific laser (Q-S / ps) | 80% |
| Tranexamic Acid (Oral) | 80% |
| Triple Combination Therapy – hydroquinone-based | 70% |
| Laser vascular (including IPL with vascular filter) | 70% |
| Thiamidol | 60% |
| Tranexamic Acid (Topical) | 60% |
| Azelaic Acid (20%) | 60% |
| Triple Combination Therapy – non-hydroquinone based | 60% |
| Chemical Peels superficial | 60% |
| Chemical Peels medium | 60% |
| Cysteamine | 60% |
| Pycnogenol (oral) | 60% |
| Anti-histamine (oral) | 60% |
| Fractional ablative laser | 60% |
| Hydroquinone (4%) | 50% |
| Topical Retinoids | 50% |
| Metformin | 50% |
| Kojic Acid | 40% |
| Malassezin | 40% |
| Methimazole | 40% |
| Arbutin | 40% |
| Melatonin (oral) | 40% |
| PRP | 40% |
| Iontophoresis | 40% |
| Antioxidants (e.g., Vitamin C) | 30% |
| Chemical Peels deep | 30% |
| 2MNG | 30% |
| Neotone | 30% |
| Niacinamide | 30% |
| Glutathione (oral) | 30% |
| Glutathione IV | 0% |

**Moderate/Severe Melasma Algorithm**

1st line of treatment

| Agent | Yes |
| --- | --- |
| Thiamidol | 100% |
| Triple Combination Therapy – hydroquinone-based | 100% |
| Triple Combination Therapy – non-hydroquinone based | 100% |
| Topical Retinoids | 90% |
| Azelaic Acid (10-20%) | 80% |
| Chemical Peels superficial | 80% |
| Hydroquinone (4%) | 80% |
| Tranexamic Acid (Oral) | 70% |
| Tranexamic Acid (Topical) | 70% |
| Cysteamine | 70% |
| Arbutin | 70% |
| Niacinamide | 60% |
| Microneedling (including radio frequency) | 60% |
| Kojic Acid | 50% |
| Antioxidants (e.g., Vitamin C) | 50% |
| Malassezin | 50% |
| 2MNG | 50% |
| Metformin | 40% |
| Methimazole | 40% |
| Neotone | 40% |
| Pycnogenol (oral) | 40% |
| Chemical Peels medium | 20% |
| Melatonin (oral) | 20% |
| Anti-histamine (oral) | 20% |
| Pigment specific laser (Q-S / ps) | 20% |
| Fractional non-ablative laser | 20% |
| Intradermal therapy | 20% |
| Glutathione (oral) | 20% |
| Laser vascular (including IPL with vascular filter) | 10% |
| Fractional ablative laser | 10% |
| PRP | 10% |
| Iontophoresis | 10% |
| Chemical Peels deep | 0% |
| Glutathione IV | 0% |

Moderate/Severe Melasma Algorithm

2nd line of treatment

| Agent | Yes |
| --- | --- |
| Anti-histamine (oral) | 80% |
| Chemical Peels medium | 80% |
| Chemical Peels superficial | 80% |
| Fractional non-ablative laser | 80% |
| Microneedling (including radio frequency) | 80% |
| Pigment-specific laser (Q-S / ps) | 80% |
| Pycnogenol (oral) | 80% |
| Tranexamic Acid (Oral) | 80% |
| Hydroquinone (4%) | 70% |
| Triple Combination Therapy – hydroquinone-based | 70% |
| Metformin | 70% |
| Cysteamine | 70% |
| Melatonin (oral) | 70% |
| Laser vascular / IPL with vascular filter) | 70% |
| Thiamidol | 60% |
| Azelaic Acid (10-20%) | 60% |
| Triple Combination Therapy – non-hydroquinone based | 60% |
| Antioxidants (e.g., Vitamin C) | 60% |
| Topical Retinoids | 60% |
| Arbutin | 60% |
| Intradermal therapy | 60% |
| Tranexamic Acid (Topical) | 50% |
| Kojic Acid | 40% |
| Malassezin | 40% |
| Methimazole | 40% |
| Neotone | 40% |
| Niacinamide | 40% |
| Fractional ablative laser | 40% |
| Iontophoresis | 40% |
| 2MNG | 30% |
| PRP | 30% |
| Glutathione (oral) | 20% |
| Chemical Peels deep | 10% |
| Glutathione IV | 0% |

Moderate/Severe Melasma Algorithm

3rd line of treatment

| Agent | Yes |
| --- | --- |
| Fractional non-ablative laser | 90% |
| Intradermal therapy | 90% |
| Laser vascular / IPL with vascular filter) | 90% |
| Microneedling (including radio frequency) | 90% |
| Pigment-specific laser (Q-S / ps) | 90% |
| Tranexamic Acid (Oral) | 70% |
| Chemical Peels medium | 70% |
| Anti-histamine (oral) | 70% |
| Thiamidol | 60% |
| Azelaic Acid (10-20%) | 60% |
| Triple Combination Therapy – hydroquinone-based | 60% |
| Cysteamine | 60% |
| Pycnogenol (oral) | 60% |
| Hydroquinone (4%) | 50% |
| Tranexamic Acid (Topical) | 50% |
| Triple Combination Therapy – non-hydroquinone based | 50% |
| Antioxidants (e.g., Vitamin C) | 50% |
| Topical Retinoids | 50% |
| Chemical Peels superficial | 50% |
| Metformin | 50% |
| Arbutin | 50% |
| Melatonin (oral) | 50% |
| Fractional ablative laser | 50% |
| PRP | 50% |
| Iontophoresis | 50% |
| Kojic Acid | 40% |
| Niacinamide | 40% |
| Malassezin | 30% |
| Methimazole | 30% |
| 2MNG | 30% |
| Neotone | 30% |
| Glutathione (oral) | 30% |
| Chemical Peels deep | 20% |
| Glutathione IV | 0% |

| **Post-Inflammatory Hyperpigmentation (PIH)** | |
| --- | --- |
| In patients who are at higher risk of developing PIH, it is highly recommended to prepare the skin before performing procedures to prevent PIH. | AGREEMENT |
|  | 100% |
| Oral tranexamic acid should be considered as a viable option for preventing PIH. | AGREEMENT |
|  | 70% |
| Some cases of PIH do not require immediate treatment, and only require waiting. | AGREEMENT |
|  | 90% |
| In the case of treatment, topical therapy should be the first line of treatment for PIH, with procedures like lasers and peels reserved for second-line or recalcitrant cases. | AGREEMENT |
|  | 100% |
| Pre-treatment with skin-lightening agents improves the efficacy of procedural therapies for PIH. | AGREEMENT |
|  | 100% |
| The use of Q-switched Nd:YAG lasers at low doses (laser-toning) is effective for treating dermal PIH in darker skin types. | AGREEMENT |
|  | 70% |
| **Periorbital Hyperpigmentation / Dark Circles** | |
| Dermoscopy should be recommended as a routine tool for diagnosing POH. | AGREEMENT |
|  | 90% |
| Stretch tests should be recommended as a routine tool for diagnosing POH. | AGREEMENT |
|  | 90% |
| Management guidelines should include recommendations for addressing iron and B12 deficiencies, especially in populations with high prevalence. | AGREEMENT |
|  | 70% |
| Periodic dermoscopic imaging should be used for tracking treatment progress. | AGREEMENT |
|  | 80% |
| **Acquired dermal macular hyperpigmentation** | |
| Patch testing (including photopatch) is recommended for: | AGREEMENT |
| Riehl´s melanosis | 100% |
| Lichen planus pigmentosus (LPP) | 50% |
| Ashy dermatosis | 30% |
| Pre-treatment with topical or oral agents is recommended before initiating procedural treatments for LPP or Riehl’s melanosis. | AGREEMENT |
|  | 90% |
| Topical and/or systemic immune modulator drugs should be used even if there is no symptoms or clinical signs of inflammation. | AGREEMENT |
|  | 70% |
| Lasers or IPL should be avoided during the active inflammatory stage of LPP to prevent exacerbation. | AGREEMENT |
|  | 100% |
| A unified nomenclature for LPP, Riehl's melanosis, and EDP under “Acquired Dermal Macular Hyperpigmentation (ADMH)” is essential for standardizing diagnosis and management. | AGREEMENT |
|  | 100% |
| Contaminants such as allergens may play a significant role in the etiology of ADMH and should be addressed during patient evaluation. | AGREEMENT |
|  | 100% |
| **Hyperpigmentation of the folds** | |
| Assessment of higher skin types with Wood’s Lamp is recommended to exclude Erythrasma. | AGREEMENT |
|  | 100% |
| Patch testing is recommended for patients’ diagnosis. | AGREEMENT |
|  | 40% |
| Topical steroids (non-fluorinated, e.g., desonide) should be limited to short-term use only. | AGREEMENT |
|  | 100% |

**According to acne**

| Treatment | PIH (acne) | PIH (non-acne) |
| --- | --- | --- |
| Avoidance of Irritants  (e.g., retinoids close to procedures) | 90% | 90% |
| Skin Priming with Lightening Agents | 80% | 100% |

| Treatment | PIH (acne) | PIH (non-acne) |
| --- | --- | --- |
| Thiamidol | 100% | 90% |
| Azelaic acid | 100% | 90% |
| Chemical Peels  (e.g., glycolic acid, TCA) | 100% | 90% |
| Topical Retinoids | 100% | 80% |
| Hydroquinone | 80% | 100% |
| Tranexamic Acid (Topical) | 80% | 80% |
| Low fluence Q-switched Nd:YAG Laser | 70% | 80% |
| Cysteamine | 60% | 80% |
| Triple combination | 50% | 80% |
| 2MNG | 50% | 60% |
| Fractional laser for drug delivery | 40% | 40% |
| Tranexamic Acid (Oral) | 30% | 80% |
| Intradermal therapy | 30% | 60% |
| Viniferin | 30% | 40% |

**Post-inflammatory Hiperpigmentation Treatment Options**

**Post-inflammatory Hiperpigmentation Prevention Options**

Topical Treatment Agents for Periorbital Hyperpigmentation

| Treatment | Constitutional Type | Post-Inflammatory Type | Vascular Type | Shadow Effect Type |
| --- | --- | --- | --- | --- |
| Azelaic Acid (10-20%) | 80% | 100% | 20% | 10% |
| Thiamidol | 80% | 90% | 30% | 30% |
| Vitamin C (L-ascorbic acid) | 70% | 100% | 80% | 60% |
| Arbutin (3%) | 70% | 90% | 10% | 10% |
| Hydroquinone (2-4%) | 70% | 90% | 0% | 0% |
| Niacinamide (Vitamin B3) | 70% | 80% | 30% | 20% |
| Retinoids (e.g., tretinoin) | 70% | 70% | 40% | 50% |
| Polyphenols | 60% | 90% | 50% | 30% |
| Kojic Acid | 60% | 70% | 10% | 10% |
| Beta-Carotene | 40% | 60% | 50% | 40% |
| Curcumin | 30% | 60% | 40% | 20% |
| Yeast Derivatives | 30% | 40% | 10% | 20% |
| Topical yeast | 20% | 20% | 10% | 10% |
| Caffeine | 10% | 10% | 100% | 10% |

**Procedural Treatments for Periorbital Hyperpigmentation**

| Treatment | Constitutional Type | Post-Inflammatory Type | Vascular Type | Shadow Effect Type |
| --- | --- | --- | --- | --- |
| Camouflage | 100% | 100% | 100% | 90% |
| Chemical Peels (e.g., glycolic acid) | 90% | 90% | 10% | 40% |
| Laser Therapy | 90% | 60% | 90% | 40% |
| Microneedling | 60% | 70% | 40% | 70% |
| Radiofrequency (RF) Therapy | 60% | 30% | 30% | 60% |
| Platelet-Rich Plasma (PRP) | 50% | 30% | 20% | 30% |
| Carboxytherapy | 30% | 20% | 40% | 40% |
| Dermal Fillers (Hyaluronic Acid) | 30% | 10% | 10% | 90% |
| Nitrogen Plasma Skin Regeneration | 10% | 20% | 10% | 20% |

**Acquired dermal macular hyperpigmentation**

| Treatment | Riehl´s melanosis | Lichen planus pigmentosus | Erythema Dyschromicum Perstans |
| --- | --- | --- | --- |
| Topical Calcineurin Inhibitors (e.g., Tacrolimus) | 100% | 100% | 78% |
| Oral isotretinoin | 90% | 100% | 90% |
| Topical Steroids | 78% | 100% | 78% |
| Lasers (e.g., Q-switched Nd:YAG, IPL) | 78% | 78% | 78% |
| Oral steroids | 67% | 100% | 78% |
| Topical JAK Inhibitor | 56% | 89% | 56% |
| Oral Tranexamic Acid | 56% | 67% | 44% |
| Dapsone | 44% | 44% | 44% |
| Oral JAK Inhibitor | 33% | 67% | 56% |
| Colchicine | 33% | 44% | 33% |
| Hydroxychloroquine | 22% | 100% | 78% |
| Cyclosporine | 22% | 67% | 44% |
| MMF | 22% | 56% | 33% |
| Clofazimine | 22% | 44% | 56% |
| Narrow-band UV | 11% | 33% | 44% |
| Chemical Peels (e.g., glycolic acid) | 11% | 33% | 22% |

**Hyperpigmentation of the folds Treatment Options**

|  |  |
| --- | --- |
| Treatment | Recommendation |
| Thiamidol | 100% |
| Niacinamide | 100% |
| Superficial peelings | 89% |
| Topical Retinoids | 90% |
| Hydroquinone | 80% |
| Desonide (0.05%) | 90% |
| Glycolic Acid (10 to 20%) and Urea (20%) | 90% |
| Q-switched Nd:YAG Laser | 90% |
| IPL (Intense Pulsed Light) | 70% |

**Hyperpigmentation of the folds Prevention Options**

| Treatment | Recommendation |
| --- | --- |
| Avoidance of Irritants and allergens | 100% |
| Avoidance trauma inducing procedures (waxing, tweezing) | 100% |
